# Abundance of Soil-Borne Entomopathogenic Fungi in Organic and Conventional Fields in the Midwestern USA with an Emphasis on the Effect of Herbicides and Fungicides on Fungal Persistence

**DOI:** 10.1371/journal.pone.0133613

**Published:** 2015-07-20

**Authors:** Eric H. Clifton, Stefan T. Jaronski, Erin W. Hodgson, Aaron J. Gassmann

**Affiliations:** 1 Department of Entomology, Iowa State University, Ames, Iowa, United States of America; 2 USDA, Northern Plains Agricultural Research Lab, Sidney, Montana, United States of America; Leibniz-Institute of Vegetable and Ornamental Crops, GERMANY

## Abstract

Entomopathogenic fungi (EPF) are widespread in agricultural fields and help suppress crop pests. These natural enemies may be hindered by certain agronomic practices associated with conventional agriculture including the use of pesticides. We tested whether the abundance of EPF differed between organic and conventional fields, and whether specific cropping practices and soil properties were correlated with their abundance. In one year of the survey, soil from organic fields and accompanying margins had significantly more EPF than conventional fields and accompanying margins. Regression analysis revealed that the percentage of silt and the application of organic fertilizer were positively correlated with EPF abundance; but nitrogen concentration, tillage, conventional fields, and margins of conventional fields were negatively correlated with EPF abundance. A greenhouse experiment in which fungicides and herbicides were applied to the soil surface showed no significant effect on EPF. Though organic fields were perceived to be more suitable environments for EPF, abiotic factors and cropping practices such as tillage may have greater impacts on the abundance of EPF. Also, fungicides and herbicides may not be as toxic to soil-borne EPF as originally thought.

## Introduction

The amount of land devoted to organic agriculture has increased from 11 million hectares in 1999 to 37.2 million hectares in 2011 [1, 2]. The National Organic Program (NOP), a regulatory program in the Agricultural Marketing Service branch of the United States Department of Agriculture, defines organic production as a system that integrates cultural, biological and mechanical practices to foster cycling of resources, promote ecological balance, and conserve biodiversity [3]. Supporting this NOP definition, meta-analyses have found that organic farms promote ecosystem services with greater evenness and abundance of natural enemies than conventional farms [1, 4–6].

One group of natural enemies that are ubiquitous in soil and on phylloplanes are entomopathogenic fungi (EPF), in particular the Ascomycetes, *Beauveria* spp. (Hypocreales: Cordycipitacea) and *Metarhizium* spp. (Hypocreales: Clavicipitaceae) [7, 8]. Entomopathogenic fungi infect aboveground and belowground insects, and they can be recovered from plant tissues and soil; however, EPF use the soil as a habitat for long-term persistence when crops are not present in the field [9]. In addition to suppressing pests, EPF may also stimulate plant growth and impede phytopathogens [7, 10]. Though a great deal of work has measured EPF in agroecosystems, more holistic research is required to determine how conventional agronomic practices, especially pesticides that are regularly applied to soil and crops, could affect the abundance of EPF in these agroecosystems, and thus, how EPF may be bolstered through conservation biological control.

A study by Klingen et al. [11] concluded that soils of organic fields had significantly more EPF than conventional fields; however, they found no significant difference between the soils from undisturbed field margins of the two cropping systems. Although data were not collected on soil properties and management practices, Klingen et al. [11] argued that organic fields are more suitable environments for EPF due to a lack of synthetic inputs and the use of organic fertilizers. A similar field study by Meyling et al. [12] did not find a difference in EPF abundance between organic and conventional cropping systems. Jabbour and Barbercheck [13] measured EPF in a field transitioning to organic production and found that EPF abundance was associated with soil properties including metallic ions, gravimetric water content and organic matter. Cropping practices that are not exclusive to organic or conventional producers, particularly tillage, could also impact EPF. Previous studies have found that cornfields and soybean fields with reduced soil disturbance had greater abundance of soil-borne entomopathogens [14–16].

There is also concern that synthetic pesticides used in cropping systems may harm EPF. Numerous studies have found that fungicides, and in some instances herbicides, can significantly reduce the germination and development of EPF [17–19]. However, most of these experiments have used *in vitro* methods where the pesticides come into direct contact with fungi developing on growth media. Experiments with fungicide-treated field plots resulted in lower EPF abundance and reduced infections of bait insects than untreated plots in some instances, but the pattern was not consistent across years and treatments [20, 21]. More *in situ* studies that attempt to simulate field scenarios are needed in order to explain the possible interactions between EPF and pesticides in agroecosystems [22].

The objectives of this study were the following: 1) within Iowa, USA determine whether or not organically farmed soils have a greater abundance of EPF than conventionally farmed soils in Iowa; 2) determine whether or not soil properties and farming practices used in these fields were correlated with the abundance of EPF; and 3) perform *in situ* greenhouse experiments to determine whether some pesticides have a deleterious effect on EPF abundance in soil. We expected to find variation in the abundance of EPF and to find that certain soil parameters and farming practices could explain some of this variation. We also expected to find a measurable decline in soil-borne EPF after application of some pesticides in the greenhouse experiment.

## Materials and Methods

### Field Study

#### Soil Sampling

**Fig 1:**
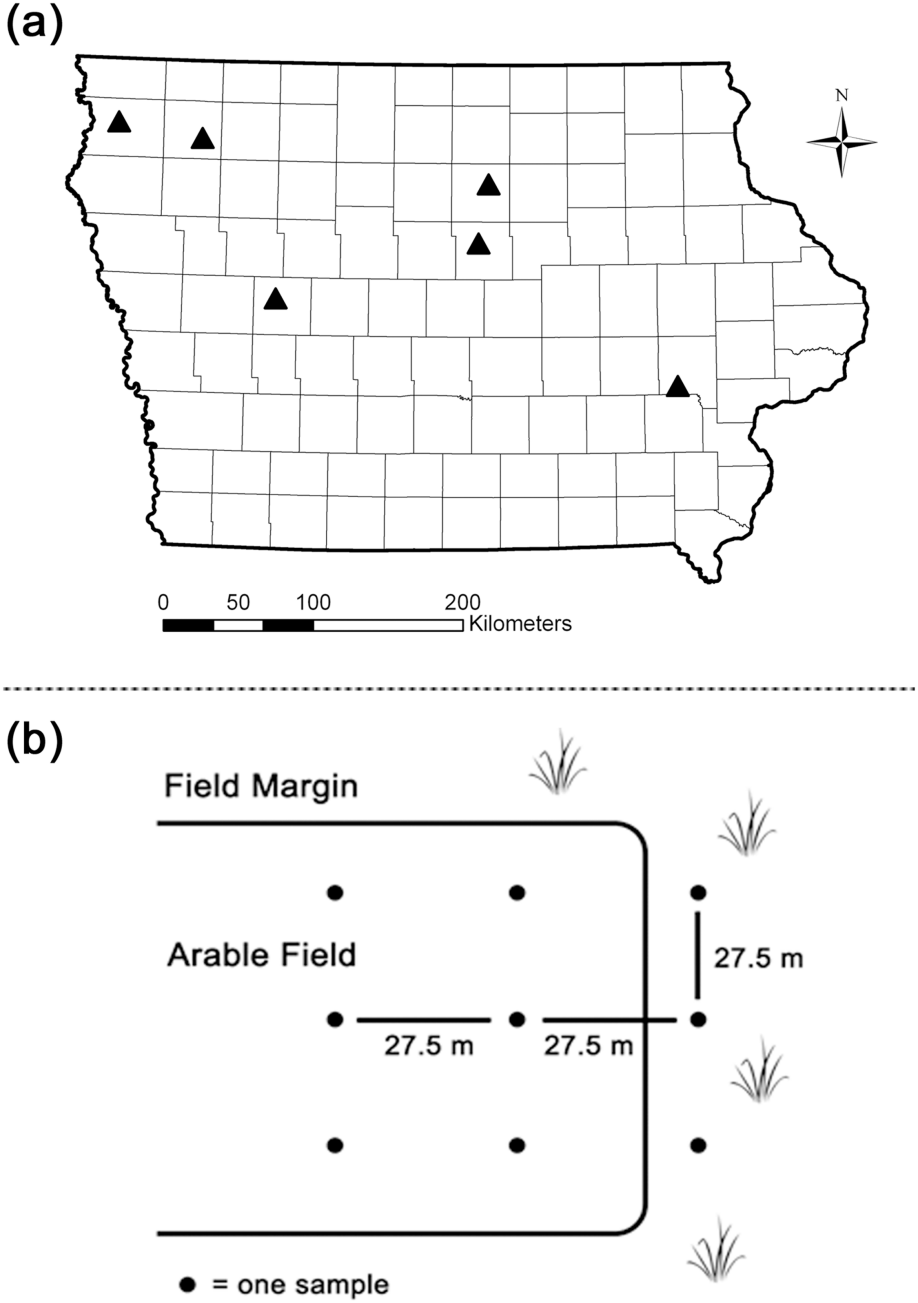
Locations sampled in Iowa during in 2011 and 2012 (a), and sampling scheme within a field (b). In (a) locations sampled, triangles indicate sites where fields were sampled, and the sampling scheme (b) illustrates how a field and accompanying margin were sampled.

We sampled six locations in Iowa in 2011 and 2012 (Fig 1a). Each location had two fields with at least three consecutive years of USDA organic certification and two conventionally farmed fields within a 5-km radius. Land owners at every farm gave us permission to sample soil and to conduct experiments with their soil. At each location, one organic and one conventional field produced corn (*Zea mays*), while the other organic and conventional fields were planted to soybean (*Glycine max*). Thus, in 2011 we sampled a total of 12 organic fields and 12 conventional fields. In 2012, we sampled the same 12 conventional fields but were only able to sample eight of the organic fields used in 2011 because four of the fields were rotated to oats (*Avena sativa*) or cattle pasture; thus, a total of 20 fields were sampled in 2012. Soil was sampled between 6–15 July 2011 and 25–30 June 2012.

After removing surface litter, soil was collected by using a cylindrical sampling tool (diameter = 10.80 cm; volume = 1 L; depth = 20 cm, Model 1001–19, Par Aide, Lino Lakes, Minnesota). The sampling tool was sterilized with 85% ethanol between samples. Within each field, three samples were collected from the field margin and six samples were collected from the interior portion of the field, with 27.5 m between each sample (Fig 1b). The field margins in our study consisted of grass strips as defined by Marshall and Moonen [23], which were generally composed of maintained brome grasses or natural vegetation in close proximity to the field crops. Samples were placed in 3.8-L polyethylene bags, returned to the lab, and stored at 5°C before use in experiments.

#### Quantifying Entomopathogen Titers by Galleria Baiting

Field soil was filtered with a 5.6-mm mesh copper sieve (Cat. no. 048815A, Fisher Scientific, Hanover Park, Illinois) to remove debris, and 150 mL aliquots of sifted soil were placed in sealable 0.35-L plastic containers and moistened to field capacity with deionized water following Goettel and Inglis [24]. Isolates of EPF were obtained by exposing third-instar larvae from a laboratory colony of the greater wax moth, *Galleria mellonella* (Lepidoptera: Pyralidae), to field soil following Goettel and Inglis [24]. Six larvae were placed in each container and held in an incubator (27°C; continuous darkness; 40% RH) for 7 days. The incubation temperature of 27°C was used in order recover both *Beauveria* spp. and *Metarhizium* spp. which have optimal growing temperatures of 25–30°C and 23–28°C, respectively [25, 26]. Containers were briefly inverted every day and returned to upright position in order to encourage the larvae to move through soil.

Dead larvae were removed from containers daily for a period of 7 days and surface-sterilized by immersion in 85% ethanol for 10 seconds before being placed in a sealed Petri dish following the methods of Goettel and Inglis [24]. Sealed Petri dishes were composed of the bottom half of a 4-cm Petri dish, lined with moistened filter paper, floating in a closed 5.5 cm Petri dish containing approximately 2 mL of deionized water. This humid environment promoted growth and sporulation of EPF conidia on cadavers. Petri dishes with insect cadavers were kept in darkness at room temperature (ca. 25°C). Cadavers were monitored for 14 days for sporulation of EPF on the cuticle. Fungi were identified by the characteristic morphology of sporulation structures with a dissecting microscope (40X magnification) and conidial shape with light microscopy (400X magnification) following Goettel and Inglis [24]. Because we did not use molecular techniques to identify fungal species, *Metarhizium anisopliae* and *Beauveria bassiana* are defined sensu lato (abbreviation “s.l.”) throughout the present study due to recent revisions to the phylogeny of these genera based on DNA sequence-based studies [27, 28]. It is possible that several different species of *Metarhizium* or *Beauveria* were recovered from insect cadavers and soil samples, and so we will refer to these fungi as *Metarhizium anisopliae* s.l. and *Beauveria bassiana* s.l. from this point forward.

#### Quantitative Enumeration via Serial Soil Dilution Plating

For each arable field, 20 g of soil from each of the six samples were combined in a 0.95-L polyethylene bag and mixed with a steel spatula for 1 min. Similarly for each field margin, 20 g of soil from each of the three samples were combined and mixed. These combined samples were then used for serial dilutions followed by plating on selective agar media to quantify EPF titers [24].

Two serial dilutions were made (1:100 and 1:1000) of each sample with a solution of autoclaved 0.10% sorbitan mono-oleate surfactant (Tween 80 Acros Organics, Morris Plains, New Jersey). For both *M*. *anisopliae* s.l. and *B*. *bassiana* s.l. three replicates of both dilutions were spread on separate 10-cm Petri dishes containing oatmeal dodine media modified from Chase et al. [29]. The medium, selective for *B*. *bassiana* s.l., has a dodine (Syllit 65W, Platte Chemical Inc., Greenville, Mississippi) concentration of 0.62 g L^-1^ and 10 mg L^-1^ crystal violet. To isolate *M*. *anisopliae* s.l., the selective medium was adjusted to a dodine concentration of 0.39 g L^-1^ and the original antibiotics were replaced with 0.25 g L^-1^ chloramphenicol.

Petri dishes were placed in a dark incubator (27°C), and checked for growth of individual colonies after 11 days, with each colony of *M*. *anisopliae* s.l. or *B*. *bassiana* s.l. scored as a single colony forming unit (CFU) using a digital pen counter. Contaminant CFUs were not quantified. The number of CFUs g^-1^ dry soil was calculated after adjusting for moisture content, which was determined gravimetrically on a parallel sample [24]. We quantified CFUs for 576 Petri dishes in 2011 and for 480 Petri dishes in 2012.

#### Soil Analysis

For each arable field, a pooled soil sample was generated by combining 20 g dried soil from each of the six samples (Fig 1b) into a polyethylene bag and mixed with a sterile steel spatula for 1 min. For a field’s accompanying margin, a pooled soil sample was generated in the same fashion, but with 20 g dried soil from each of the three field margin samples (Fig 1b). These pooled samples were submitted to the Iowa State University Soil and Plant Analysis Laboratory to obtain percent organic carbon and nitrogen by combustion methods following Combs and Nathan [30]. Soil samples also were sent to the Iowa State University Agronomy Department to obtain percent sand, clay, and silt composition following the particle size analysis protocol of Gee and Bauder [31].

#### Field History

Farmers were asked a standardized set of questions each year regarding farming practices applied that year prior to the time when a field was sampled.

1. Was the field cultivated at least once?
2. Was the field fertilized?
3. If fertilized, was an organic (i.e., followed the definitions of allowed substances in organic crop production [3]) or synthetic fertilizer used?
4. Was the field sprayed with herbicide?
5. Was the field sprayed with fungicide?

### Pesticide Experiment

This experiment was conducted between 5 April and 8 June, 2012, and consisted of four fungal treatments fully crossed with five pesticide treatments, for a total of 20 treatments. For fungal treatments cups of soil were either inoculated with one of the three *Metarhizium* strains or served as a control that did not receive inoculation with fungi. For each of the four fungal treatments, five cups of soil were prepared and received one of the five pesticide treatments that consisted of either one of the four pesticides or a control that lacked pesticides. This experimental design was defined as one block and a total of eight blocks were established for a total of 160 containers of soil. A block was setup every six days during the experiment.

We used three different strains of *Metarhizium* spp. (i.e., *Metarhizium anisopliae* sensu lato): *Metarhizium robertsii* DWR346 (= USDA ARS Entomopathogenic Fungus Collection, ARSEF 8367), *Metarhizium robertsii* DWR356 (= ARSEF 9621) and *Metarhizium anisopliae* MA1200 (= ARSEF 6958). The two *M*. *robertsii* isolates were derived from soils in Arizona and Utah, respectively, plated on selective media and MA1200 was from Illinois and was isolated from the egg of a soybean cyst nematode, *Heterodera glycines* (Tylenchoidea: Heteroderidae) [32]. We used strains from diverse geographic locations and sources of collection to capture more of the genetic diversity present within the genus. Conidia were produced with solid substrate fermentation following Jaronski and Jackson [33]. Conidial titers in each spore powder were determined by hemocytometer count of diluted conidial suspensions. Viability of each strain was determined 24 h prior to soil inoculations via germination on sabouraud dextrose agar after 18 h of incubation at 27°C [24].

Soil used in the experiment was obtained from a conventionally-managed field in corn and soybean rotation located on an Iowa State University research farm in Ames, Iowa. Soil was analyzed following the same methods used in the field survey and found to be composed of 40.9% sand, 23.1% silt and 36.0% clay. Prior to use, soil was air dried and sieved through a 1-mm mesh sieve. The water holding capacity (WHC) was determined gravimetrically following Gardner [34]. For the each inoculation, ca. 1600 g dry soil was inoculated with a single strain of *Metarhizium* spp. using a conidial suspension that contained 4.0x10^6^ conidia mL^-1^. Inoculations for the control that did contain fungi were suspensions of autoclaved 0.10% sorbitan mono-oleate. Inoculations were done at a rate of 0.31 mL g^-1^ dry soil and this produced a soil mixture at ca. 25% WHC containing 1.0x10^6^ conidia g^-1^ soil. Conidial suspensions were made in autoclaved 0.10% sorbitan mono-oleate and were incorporated into soil using a sterile spatula. Approximately 250 mL of the inoculated soil was placed in the 0.35-L polyethylene cups and then weighed. After inoculation (day 0), cups were randomly arranged on a greenhouse bench that was 60 cm below 400 W high-pressure sodium bulbs (Ruud Lighting Inc., Racine, Wisconsin). The greenhouse was kept on a 14:10 day:night cycle with a temperature range of 24°C to 32°C and humidity of ca. 30%. Every 2 days, deionized water was added to return soil to 25% WHC based on the original mass of each cup.

Pesticides were applied at the highest allowable field rate for a one-time application to corn and soybean. The herbicides tested were glyphosate (RoundUp PowerMax; Monsanto Co., St. Louis, MO) and glufosinate ammonium (Ignite, Bayer CropScience, Monheim, Germany), and these were applied at rates of 1608 mL/ha (2086 mg active ingredient/L) and 2631 mL/ha (1457 mg active ingredient/L), respectively. The fungicides tested were a mixture of prothioconazole and trifloxystrobin (Stratego YLD, Bayer CropScience), which was applied at 365 mL/ha (91 mg prothioconazole/L and 271 mg trifloxystrobin/L); and pyraclostrobin (Headline, BASF, Ludwigshafen, Germany), which was applied at 877 mL/ha (435 mg active ingredient/L). Fungicides were mixed with a solution of 0.10% crop oil (Prime Oil, Winfield Solutions, Minneapolis, Minnesota) and herbicides were mixed with deionized water.

On day eight of the experiment, containers of soil were treated with one of four different pesticides or a control solution 0.10% crop oil. Pesticides were applied to cups using a motorized DeVries spray chamber with a single flat spray tip (80015-VS, TeeJet Technologies, Wheaton, Illinois). All of the pesticides were applied 30 cm above cups at a rate of 168 L hectare^-1^ (after mixing with deionized water or 0.10% crop oil carrier). Cups of soil were returned to the greenhouse bench and moistened to 25% WHC every 48 h with deionized water for the duration of the experiment.

On day 15, CFUs were quantified by sampling soil from the center of each cup, and conducting serial dilutions in the same manner as the field survey. For each cup, 10 g of soil was removed from the center and crushed before using 1 g in serial dilutions. Three aliquots for both the 1:100 and 1:1000 dilutions were plated on selective media. We counted CFUs on six Petri dishes from each of the eight cups used for each combination of four fungal treatments by five pesticide treatments, for a total of 960 Petri dishes plated from 160 cups. The 1:100 dilutions produced more consistent numbers of CFUs (20–100 per Petri dish), thus the 480 Petri dishes from the 1:100 dilutions were used for data analysis. The three aliquots in every 1:100 dilution CFUs for each of the eight cups per treatment.

In a similar fashion to the field study, we used larval *G*. *mellonella* to detect EPF. After performing serial dilutions on day 15, five 44-mL polystyrene containers with lids (Model P150-0100, Solo Cup Company, Highland Park, Illinois) were prepared from each of the replications from the greenhouse portion of the experiment. One *G*. *mellonella* larva and 10 g of crushed soil were placed in each 44 mL container and held in a dark cabinet at room temperature (ca. 24°C) for 7 days. Dead insects were removed daily and placed on Petri dishes with moistened filter paper in the same fashion as described in the field study. Sporulating cadavers were identified based on the characteristic morphology of *Metarhizium*. The proportion of *G*. *mellonella* larvae killed after 7 days for each cup of soil was recorded.

### Data analysis

Unless otherwise stated, all data analyses were conducted with SAS Enterprise Guide 5.1 [35]. For the field survey, mortality of *G*. *mellonella* was coded as a binomial variable, with each cup scored as either positive or negative for at least one larvae killed by either *B*. *bassiana* s.l. or *M*. *anisopliae* s.l. (none of the cups contained both larvae killed by *B*. *bassiana* s.l. and larvae killed by *M*. *anisopliae* s.l.) Data on number of cups that were positive *vs*. negative for EPF (either *B*. *bassiana* s.l. or *M*. *anisopliae* s.l.) were transformed by the function x + 1 to enable analysis with a test of independence based on a log-linear model (PROC CATMOD) that included the factors of farming practice (conventional *vs*. organic), crop (corn *vs*. soybean), and treatment (arable field *vs*. margin).

In both years of the field study, only two organic fields produced CFUs for *B*. *bassiana* s.l. colonies on selective media, and as a result, these data were not analyzed. Data on density of *M*. *anisopliae* s.l. CFUs in the field survey were analyzed with a mixed-model analysis of variance (ANOVA) (PROC MIXED), and data were analyzed separately for each year. The fixed effects were practice (organic *vs*. conventional), crop (corn *vs*. soybean), and treatment (arable field *vs*. margin). The random effects were site, which was the six different sites in Iowa from which soil was sampled, and all possible interactions of site with fixed effects. Random effects were tested using a log-likelihood ratio statistic (-2 RES log likelihood in PROC MIXED) based on a one-tailed χ^2^ test assuming one degree of freedom [36]. Random factors were removed from the model to increase the statistical power when these factors were not significant at a level of α < 0.20 [37]. The numbers of *M*. *anisopliae* s.l. CFUs g^-1^ soil were log transformed to normalize residuals.

Data on density of *M*. *anisopliae* s.l. CFUs in the field survey were also analyzed with multiple regression analysis (PROC REG) using forward and backward stepwise selection, (SELECTION = STEPWISE), with *P* > 0.15 for exclusion from the model and *P* < 0.15 for inclusion in the model [36]. Data were analyzed separately for 2011 and 2012 and were log transformed. The factors used in the regression analysis included: percentage values for sand, silt, clay, organic C, and total N; binomial variables for applications of herbicide, fungicide, synthetic fertilizer, organic fertilizer, and tillage; and the two by two factorial combinations of practice (organic *vs*. conventional) by treatment (arable field *vs*. field margin).

We used a mixed model ANOVA to analyze data in the pesticide experiment, with CFUs after pesticide application as the dependent variable, and the fungal treatment, pesticide treatment and the interaction of these two factors as fixed effects. The random effects included block and all interactions of block with the fixed effects. The same fixed and random effects were used for a mixed model ANOVA to analyze the proportion of *Galleria mellonella* killed by EPF. When a significant effect was present pairwise comparisons were made using the PDIFF statement in PROC MIXED with a Bonferroni adjustment for multiple comparisons.

## Results

**Fig 2:**
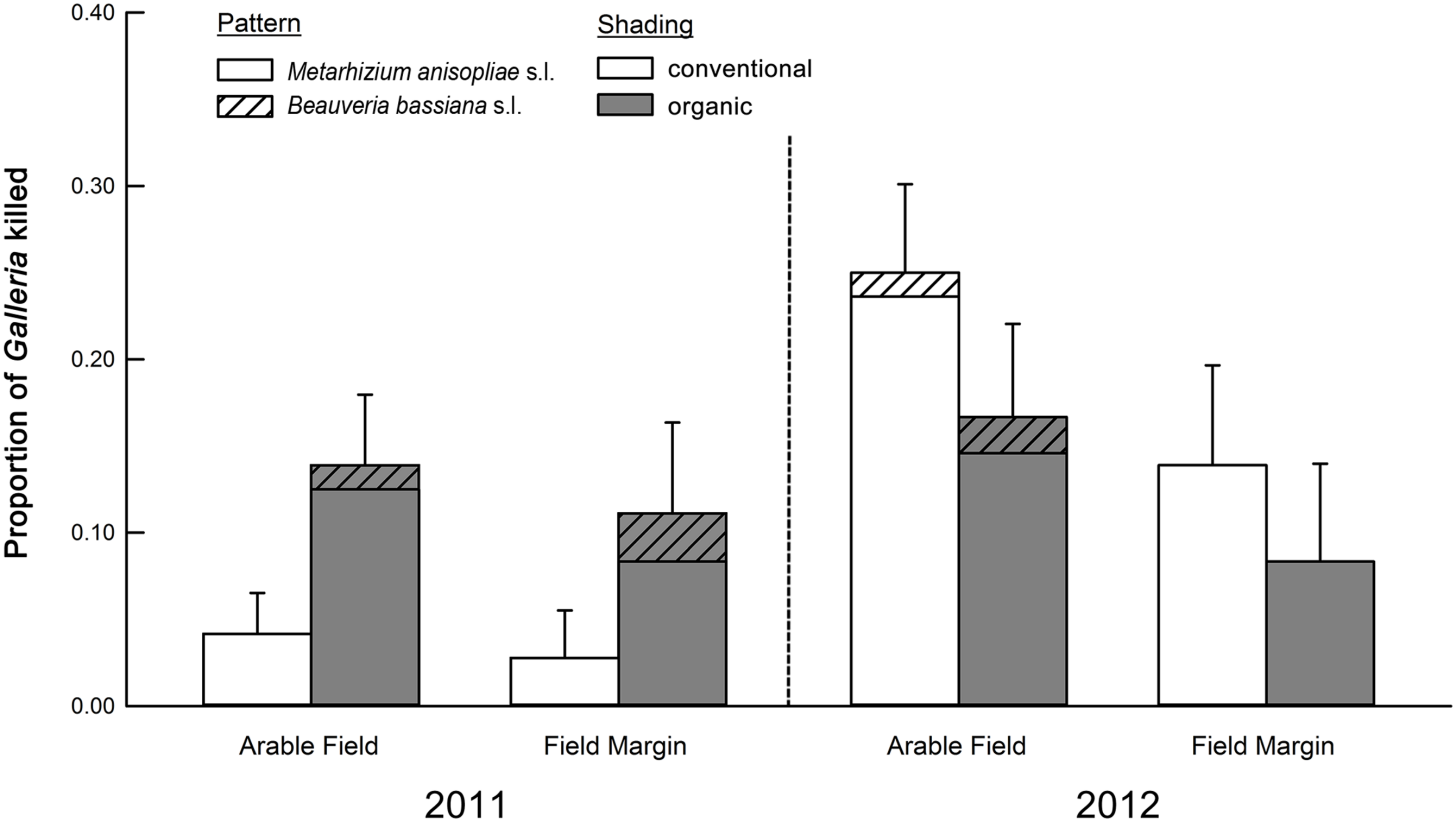
Proportional mortality of *Galleria mellonella* from *Metarhizium anisopliae* s.l. and *Beauveria bassiana* s.l. Bar heights represent sample means and error bars are the standard error of the mean.

**Fig 3:**
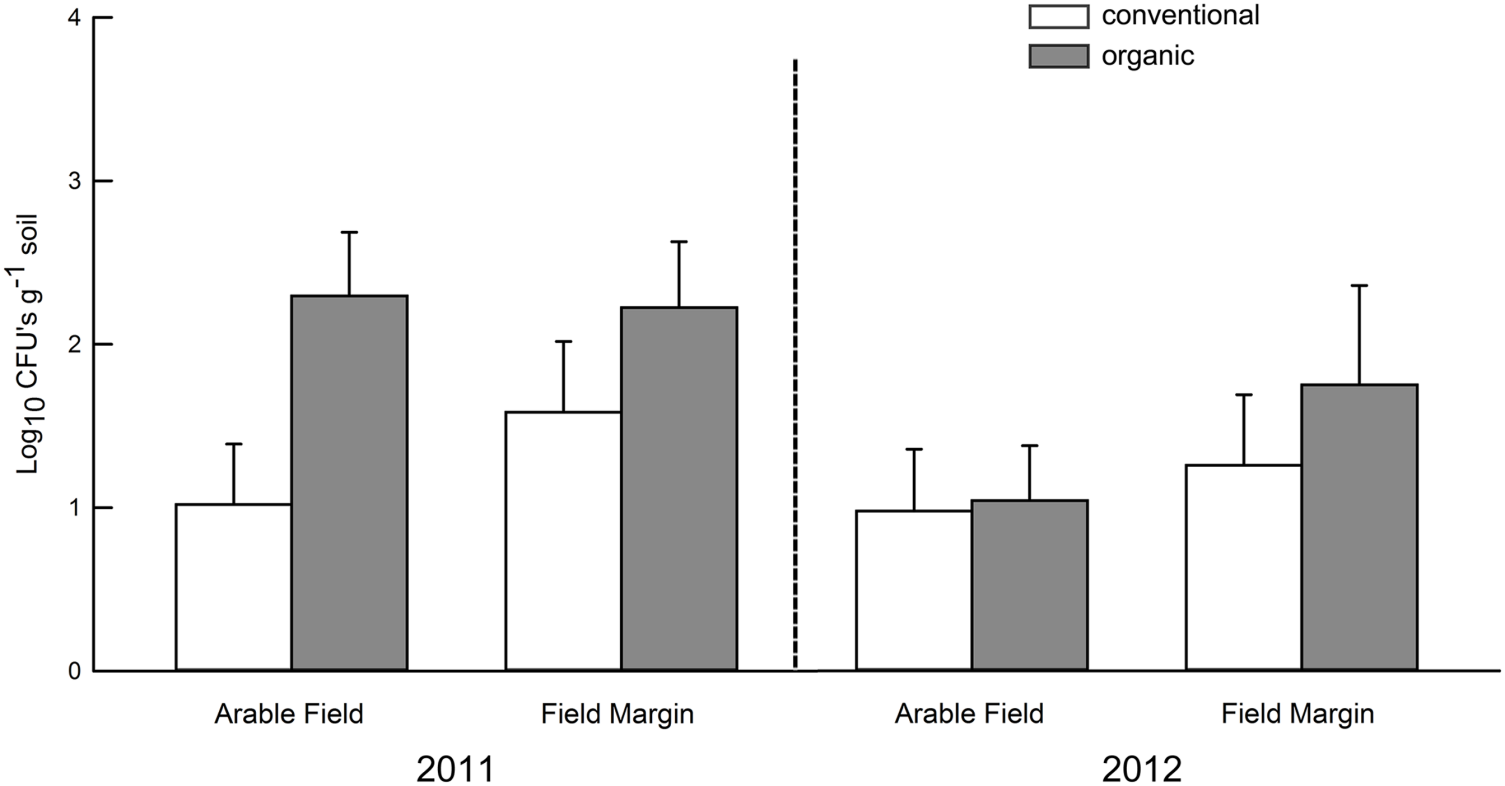
Abundance of colony forming units (CFUs) of *Metarhizium anisopliae* s.l. in soil. Bar heights are sample means and error bars are the standard error of the mean.

Based on mortality of *G*. *mellonella* from EPF, there was significantly greater occurrence of EPF in organically farmed soil than conventionally farmed soil in 2011 (Fig 2; Table 1). However, in 2012 no statistically significant differences were detected (Table 1). Additionally, in 2011, abundance of *M*. *anisopliae* s.l. CFUs in organic agroecosystems was significantly higher than conventional agroecosystems (Fig 3; Table 2). However, no significant differences were detected in 2012 (Table 2).

**Table 1:** Test of independence for mortality of *Galleria mellonella* by *Metarhizium anisopliae* s.l. or *Beauveria bassiana* s.l.

Year	Source	df^*a*^	χ^2^	*P*
**2011**	Practice^*b*^	1	3.84	0.05
	Crop^*c*^	1	0.47	0.49
	Treatment^*d*^	1	0.12	0.73
	Practice × Crop	1	2.23	0.13
	Practice × Treatment	1	0.09	0.76
	Crop × Treatment	1	<0.01	0.96
	Practice × Crop × Treatment	1	0.57	0.45
**2012**	Practice	1	0.20	0.66
	Crop	1	1.95	0.16
	Treatment	1	0.68	0.41
	Practice × Crop	1	2.59	0.11
	Practice × Treatment	1	<0.01	0.95
	Crop × Treatment	1	0.57	0.45
	Practice × Crop × Treatment	1	0.11	0.74

^*a*^ df: degrees of freedom

^*b*^ Practice: organic vs conventional

^*c*^ Crop: corn vs soybean

^*d*^ Treatment: arable field vs field margin

**Table 2:** Analysis of variance for the number of colony forming units (CFUs) of *Metarhizium anisopliae* s.l. in the field study.

Year	Source	df^*a*^	*F*	*P*
**2011**	Practice^*b*^	1, 35	6.55	0.01
	Crop^*c*^	1, 35	1.74	0.20
	Treatment^*d*^	1, 35	0.43	0.52
	Practice × Crop	1, 35	0.08	0.78
	Practice × Treatment	1, 35	0.72	0.40
	Crop × Treatment	1, 35	1.92	0.17
	Practice × Crop × Treatment	1, 35	0.05	0.83
**2012**	Practice	1, 27	0.34	0.57
	Crop	1, 27	0.51	0.48
	Treatment	1, 27	0.80	0.38
	Practice × Crop	1, 27	0.47	0.50
	Practice × Treatment	1, 27	0.09	0.77
	Crop × Treatment	1, 27	0.58	0.45
	Practice × Crop × Treatment	1, 27	0.19	0.67

^*a*^ df: numerator degrees of freedom, denominator degrees of freedom

^*b*^ Practice: organic vs conventional

^*c*^ Crop: corn vs soybean

^*d*^ Treatment: arable field vs field margin

Multiple regression analysis of *M*. *anisopliae* s.l. CFU abundance in 2011 explained 23% of the variation and retained the parameters of percent nitrogen, tillage, conventional field and herbicide application, conventional margin, percent silt, and organic fertilizer (Table 3). Conventional field and herbicide were found to be collinear parameters, with every conventional field receiving herbicide, and thus, were combined into a single parameter. The model revealed that the density of *M*. *anisopliae* s.l. CFUs was positively correlated with soils that received organic fertilizers and had higher silt composition, but was negatively correlated with nitrogen content, tillage, adjacency to conventional fields (i.e., conventional margin), and conventional fields (or the herbicides that were applied to conventional fields). For 2012, no parameters were statistically significant and thus no variation in the data could be explained with the regression analysis. A summary of the soil properties and management practices for these field and their margins can be viewed in the supplemental material (S1 and S2 Tables).

**Table 3:** Multiple linear regression for number of colony forming unit (CFU) of *Metarhizium anisopliae* s.l. in 2011.^a^

Variable	Slope	Std. error	*F*	*P*
**Total Nitrogen (%)**	-7.47744	2.87781	6.75	0.01
**Tillage**	-1.16204	0.37193	9.76	<0.01
**Conventional Field & Herbicides**	-0.89547	0.34473	6.75	0.01
**Conventional Margin**	-0.86284	0.34885	6.12	0.01
**Percent Silt**	0.02252	0.00858	6.88	0.01
**Organic Fertilizer**	1.45412	0.46996	9.57	<0.01
**(Intercept)**	2.42664	0.57335	17.91	<0.01

^a^R^2^ = 0.23

**Fig 4:**
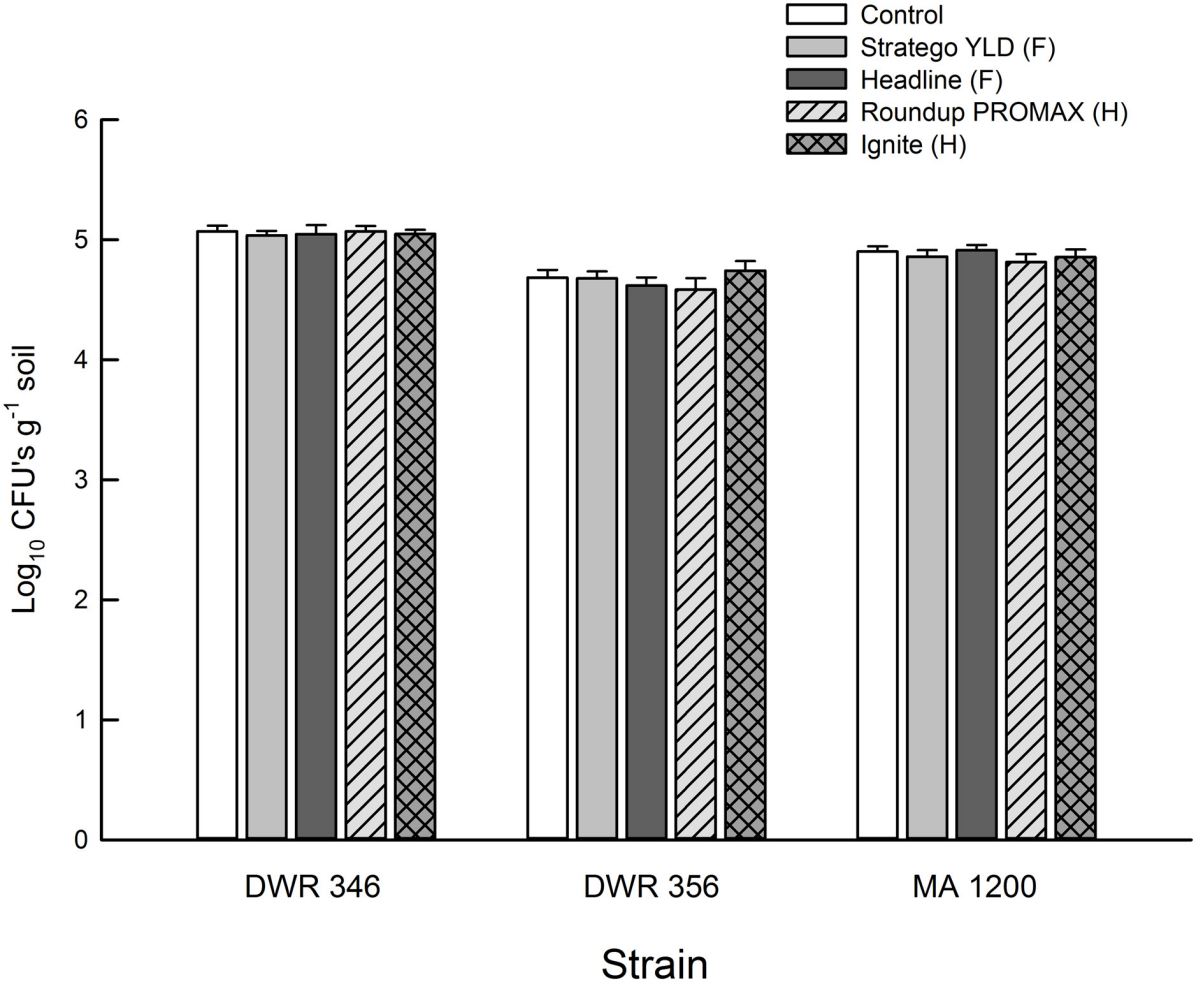
Abundance of colony forming units (CFUs) of *Metarhizium* spp. in cups of soil treated with foliar applications of fungicides (F) or herbicides (H). Bar heights are sample means and error bars are the standard error of the mean.

**Fig 5:**
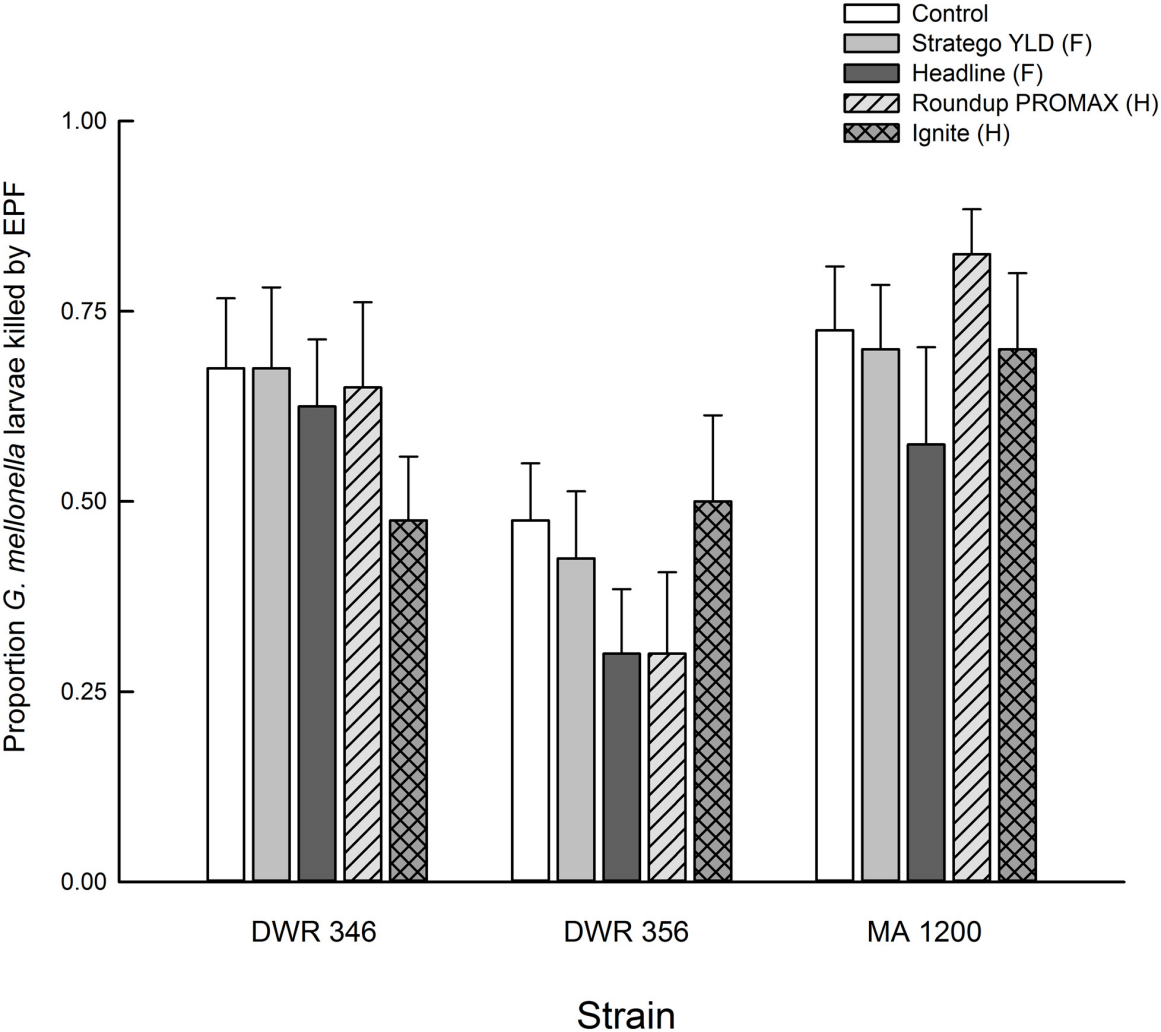
Proportional mortality of *Galleria mellonella* from *Metarhizium* spp. in cups of soil treated with foliar applications of fungicides (F) or herbicides (H). Bar heights are sample means and error bars are the standard error of the mean.

In the pesticide experiment, fungal treatment significantly affected both the final number of CFUs and mortality of *G*. *mellonella* from *Metarhizium* spp. (Figs 4 and 5, Table 4). Strain DWR 346 had overall higher CFUs than strain DWR 356 (df = 98, t-value = 12.42, *P* < 0.0001) and strain MA 1200 (df = 98, t-value = 5.42, *P* < 0.0001), and strain MA 1200 had higher CFUs than strain DWR 356 (df = 98, t-value = 7.00, *P* < 0.0001). We also found that strain DWR 356 imposed overall lower proportion mortality of *G*. *mellonella* than strain DWR 346 (df = 133, t-value = 4.93, *P* < 0.0001) and strain MA 1200 (df = 133, t-value = 6.83, *P* < 0.0001), but no difference was detected between MA 1200 and DWR 356 (df = 133, t-value = 1.90, *P* = 0.1769). However, there was no significant effect of pesticide treatment or significant interaction between pesticide treatment and fungal treatment (Table 4), indicating the exposure to fungicides and herbicides did not significantly reduce the number of viable conidia or the capacity of *Metarhizium* spp. to kill *G*. *mellonella* (Figs 4 and 5). Additionally, the dead *G*. *mellonella* from the controls that were not inoculated with *Metarhizium* spp. did not develop conidia of *Metarhizium* spp. and none of the Petri dishes receiving soil suspensions from these controls produced *Metarhizium* spp. CFUs, indicating that we did not detect any background levels of *Metarhizium* spp. in the soil we used for the experiment.

**Table 4:** Mixed model analysis of variance for effect of pesticides on *Metarhizium* spp.

Model	Effect	df	*F*	*P*
**Model #1** ^***a***^	Fungi	2, 14	19.05	<0.01
	Pesticide	4, 28	1.17	0.34
	Fungi × Pesticide	8, 56	0.90	0.53
**Model #2** ^***b***^	Fungi	2, 14	14.50	<0.01
	Pesticide	4, 28	1.31	0.29
	Fungi × Pesticide	8, 56	1.60	0.14

^*a*^ Model #1: *Metarhizium* spp. colony forming unit (CFU) g^-1^ soil

^*b*^ Model #2: Mortality of *Galleria mellonella* larvae from *Metarhizium* spp.

## Discussion

In 2011, we found that the occurrence of EPF was significantly greater in the soil of organic agroecosystems than conventional agroecosystems. This was the case for total abundance of *Beauveria bassiana* s.l. and *Metarhizium anisopliae* s.l. determined by baiting with *Galleria mellonella* (Table 1, Fig 2) and for the abundance of *M*. *anisopliae* s.l. measured by counting CFUs in soil (Table 2, Fig 3). Our findings are consistent with Klingen et al. [11] who found greater occurrence of EPF in soil of organic farms compared to conventional farms in Norway. It is important to note that the choice of bait insect can influence the diversity of EPF recovered from soils. For example, Klingen et al. [11] used *G*. *mellonella* to recover *Metarhizium* spp. and *Beauveria* spp. from soils, but also used *Delia floralis* (Diptera: Anthomyiidae) which recovered the entomopathogenic fungus *Tolypocladium cylindrosporum* (Hypocreales: Ophiocordycipitaceae). Aside from EPF, other studies have observed greater abundance of natural enemies including predatory beetles, entomopathogenic nematodes, and parasitoids within cropping systems using organic practices [38, 39]. However, this pattern of increased entomopathogen abundance may not be consistent across years, as indicated by our 2012 data, and by other multi-year studies [13, 14]. It may be the case that organic soils foster a greater abundance of soil-borne EPF, but additional research is needed to better understand the effects of organic practices on EPF in corn and soybean fields in the Midwestern United States.

Multiple regression analysis of data from 2011 showed that several factors including physical properties of soil and some cropping practices, were significantly correlated with the abundance of *M*. *anisopliae* s.l. Silt content and use of organic fertilizers were positively correlated with abundance of *M*. *anisopliae* s.l. Soils with higher silt content could have greater water retention and consequently may protect the fungal conidia from desiccation [40]. Additionally, soils that received organic fertilizer were positively associated with abundance of *M*. *anisopliae* s.l. Applications of organic fertilizer are not exclusive to organic farmers and some conventional farmers in our survey used them. Organic fertilizers may provide decomposed plant tissues as substrates for EPF mycelium and/or increase the abundance of soil-inhabiting insects and consequently increase the abundance of potential hosts available to EPF [11]. Meta-analyses showed greater invertebrate abundance and evenness in organic agroecosystems which in turn may support a greater abundance of diversity of EPF [4–6]. It is possible that our organic fields studied in 2011 had more insect activity in the soil and thereby more EPF infections compared to the conventional fields.

In 2011, several factors also were negatively correlated with abundance of *M*. *anisopliae* s.l. CFUs. Consistent with the results of ANOVA (Table 2; Fig 3), both conventional field and conventional margin showed significant negative effects in multiple regression analysis (Table 3). Because all conventional fields received at least one application of herbicide, it is unclear to what extent herbicides may have negative effects on EPF in conventional fields. Additionally, none of the farmers, organic or conventional, used fungicides in their fields before the time of soil sampling. The significant effect of conventional margin suggests that EPF in conventional field boundaries may be negatively affected by practices that occur within the field, for example, herbicide drift. de Snoo [41] observed lower abundance and lower diversity of insects inhabiting field margins that were sprayed with herbicides compared to the field margins left unsprayed. Lower numbers of insects in these reservoirs outside the field could correlate to fewer hosts for EPF [23]. Additionally, CFU abundance decreased with soil nitrogen content (Table 3). Due to the extreme complexity of soil environments, discerning the relationship between nitrogen concentrations and EPF abundance is difficult. This result could be indirectly mediated by a diversity of soil microorganisms, for example, bacteria that may exploit elevated nitrogen concentrations and subsequently outcompete EPF propagules for substrates. Tillage was also associated with lower abundance of *M*. *anisopliae* s.l. Tillage may be harmful to soil microorganisms, even in organically managed systems, because tillage can move EPF to the soil surface thereby exposing conidia to ultraviolet radiation, high temperatures, and desiccation [13, 14, 42]. Across all years of a field study, Sosa-Gomez and Moscardi [16] measured higher titers of soil-borne EPF in no-till soybean fields compared to fields that were tilled. It should be noted that there were other soil and field properties we did not measure, which likely may have explained additional variation of EPF titers. We did not ask farmers about insecticide applications and the history of insect pests in these fields. In addition, micronutrients, metallic ions, pH and other soil properties were not quantified in our field survey.

Although many studies have found herbicides and fungicides to be inhibitory to EPF *in vitro*, fewer have been able to replicate this effect in the field. Similar to Bruck [43], we found no effect of fungicides on EPF in bulk soil (Figs 4 and 5, Table 4). Furthermore, the two herbicides used in our experiment had no significant impact on EPF. Loria et al. [44] found that the fungicides mancozeb and metiram were inhibitory to *B*. *bassiana* s.l. on agar media in the laboratory, but only mancozeb negatively impacted *B*. *bassiana* s.l. abundance on potato foliage in the field. Fewer studies have measured the negative impacts of herbicides and fungicides on EPF in belowground systems. Based on our findings and previous studies, it would appear that some herbicides and fungicides do not have a significant impact on *M*. *anisopliae* s.l. in bulk soil in the short term nor do they impact infection of some host insects [43, 45]. Future studies should measure soil-borne EPF titers over more time and include the rhizosphere (i.e., plant-soil interface) to better simulate field scenarios.

The fungus *B*. *bassiana* s.l. was rarely recovered from our soil samples, but it does not necessarily mean that it is scarce in agroecosystems. A review by Scheepmaker and Butt [46] found that geometric mean fungal densities of *B*. *bassiana* s.l. varied greatly by soil type and crop, whereas geometric mean fungal titers for *M*. *anisopliae* s.l. were more cosmopolitan and greater in density. Also, *M*. *anisopliae* s.l. is easily isolated from agricultural soils whereas *B*. *bassiana* s.l. tends to be found in undisturbed forest habitats and semi-natural landscapes [47–49]. Rudeen et al. [8] recovered *B*. *bassiana* s.l. in 60% of soil samples from corn fields using the same *Galleria* bait method; however, samples were taken from corn root masses and it is possible that *B*. *bassiana* s.l. prefers to reside in close proximity to plant tissue as opposed to bulk soil. Our soil samples were taken between crop rows adjacent to crops, but plants were not uprooted. *M*. *anisopliae* s.l. CFUs in our soil samples were in the thousands per gram of soil. By contrast, for the few Petri dishes of selective media that produced *B*. *bassiana* s.l., densities were ca. 150 CFUs g^-1^ soil, and as such, other samples with *B*. *bassiana* s.l. could have gone undetected with the dilutions used on the selective media. Consistent with this hypothesis, Bing and Lewis [14] found an average of only 51 to74 *B*. *bassiana* s.l. CFUs g^-1^ soil in Iowa cornfields.

In contrast to data from 2011, the summer of 2012 showed no significant differences between organic and conventional cropping systems and this may have resulted from abnormally high seasonal temperatures and drought that occurred in Iowa during 2012. By the end of July 2012, 100% of Iowa was in severe drought; by contrast, at the end of July 2011, 0% of Iowa was in severe drought [50]. Many of the studies on EPF have concluded that environmental conditions including precipitation and temperatures can explain seasonal variations in abundance [13, 14, 51]. For example Yaginuma [52] found that rainfall varied greatly by year but was highly correlated (r = 0.94) with the number of apple orchard insects infected by EPF. The warm weather during the spring of 2012 may have shortened the date of early insect activity for populations of insect pests in Iowa fields [53]. As a consequence of the earlier insect activity in 2012, EPF titers may have been bolstered by greater numbers of invertebrate hosts that resided in the soil before our time of sampling. In addition, the extreme drought caused cracking of the upper 15 to 30 cm of most soils, and thus no-till fields may have suffered the same changes to topsoil that tilled fields experienced at the time of sampling [53]. We hypothesize that the 2012 drought masked any soil-altering effects of farming practices that may have explained the variation of EPF titers in 2011 when precipitation and temperatures were closer to the historical averages.

To preserve EPF and thereby their beneficial services, data are needed on how cultural, biological and mechanical practices in cropping systems may affect EPF. For one year of the field study, we found that factors including the particulate composition of soil, absence of tillage, and organic fertilizers were correlated with enhanced abundance of *M*. *anisopliae* s.l. The severe drought in 2012 may have negated any abundance-bolstering effects of agricultural practices like no-tillage on soil-borne EPF titers. In 2011, we found that the abundance of *M*. *anisopliae* s.l. in field margins was negatively affected by proximity to conventional fields, suggesting that cropping practices within a field could affect soil-borne microorganisms outside of a field. However, it is unclear if such negative effects would extend beyond the ecologically homogenous grassy field margins evaluated in this study. To the extent that increased EPF enhances suppression of pest insects, these data indicate that organic cropping systems will enjoy the benefit of reduced pest abundance during some years. Furthermore, crop producers may be able to take advantage of ecosystem services provided by EPF through conservation biological control by adopting practices that enhance EPF abundance, including use of organic fertilizers and no-till agriculture. At the same time, there are some factors that cannot be manipulated by growers, including soil composition and weather, which can greatly impact communities of natural enemies in belowground systems. Because of the 2012 drought and the implications of climate change, future multi-year studies should monitor communities of soil microbiota in agroecosystems and how beneficial organisms like *Metarhizium anisopliae* s.l. may be affected by weather extremes. Considering the results of both the multiple regression analysis and the pesticide experiment, soil-borne EPF appear to be robust to some conventional farming practices whereas other practices, especially tillage, could have greater impacts on EPF titers. More research will be required to quantify the benefits achieved by cultivating a higher abundance of EPF in agricultural soils.

## Supporting Information

S1 Table Field histories and soil properties for 2011 soil samples. (DOCX)

S2 Table Field histories and soil properties for 2012 soil samples. (DOCX)
