# Supplementary material for: Abundance of Soil-Borne Entomopathogenic Fungi in Organic and Conventional Fields in the Midwestern USA with an Emphasis on the Effect of Herbicides and Fungicides on Fungal Persistence
Source: PLoS One. 2015 Jul 20;10(7):e0133613. doi: 10.1371/journal.pone.0133613 (PMC4507996; doi:10.1371/journal.pone.0133613)
Supplement: S1 Table — (DOCX) [file pone.0133613.s001.docx]

**2011 Field Survey**

**Site Practice Crop Treatment CFUs*^a^* % Sand % Clay % Silt % C*^b^* % N*^c^* Tillage*^d^* Org. F Herbicides**

Iowa Falls Conventional Corn Field 3.45 44.8 22.4 32.8 2.183 0.1454 1 1 1

Iowa Falls Conventional Corn Margin 1.02 47 22.3 30.7 2.655 0.155 0 0 0

Iowa Falls Conventional Soybean Field 0.00 37.4 23.3 39.4 1.969 0.1375 0 0 1

Iowa Falls Conventional Soybean Margin 1.12 41.3 21.2 66 3.16 0.1801 0 0 0

Iowa Falls Organic Corn Field 0.74 49.5 20.4 30.1 1.404 0.1204 1 0 0

Iowa Falls Organic Corn Margin 0.00 50.2 19 30.7 1.818 0.1345 0 0 0

Iowa Falls Organic Soybean Field 0.00 32.9 25 42.1 2.458 0.1131 1 0 0

Iowa Falls Organic Soybean Margin 1.02 44.6 21.1 34.4 2.289 0.1274 0 0 0

Kalona Conventional Corn Field 1.02 3.3 18.5 78.2 1.764 0.1226 1 1 1

Kalona Conventional Corn Margin 4.01 6.6 19.1 74.3 1.918 0.1051 0 0 0

Kalona Conventional Soybean Field 3.66 3.5 17.6 78.9 1.878 0.1632 0 0 1

Kalona Conventional Soybean Margin 0.98 4 21 75 1.344 0.077 0 0 0

Kalona Organic Corn Field 2.24 3.5 19.6 76.9 1.798 0.1256 1 1 0

Kalona Organic Corn Margin 3.51 5.4 20.8 73.8 2.506 0.1366 0 0 0

Kalona Organic Soybean Field 4.03 16.5 14.8 68.7 1.467 0.0877 0 0 0

Kalona Organic Soybean Margin 3.54 12.2 16.7 71.1 2.272 0.1727 0 0 0

Hampton Conventional Corn Field 1.01 65.8 12 22.2 1.492 0.0683 1 0 1

Hampton Conventional Corn Margin 0.00 51.2 15.6 33.2 1.437 0.1074 0 0 0

Hampton Conventional Soybean Field 1.02 65.8 12 22.2 1.492 0.0683 1 0 1

Hampton Conventional Soybean Margin 2.34 48.2 21.4 30.4 4.803 0.1932 0 0 0

Hampton Organic Corn Field 2.19 47.4 19.1 33.6 1.202 0.0618 1 0 0

Hampton Organic Corn Margin 3.25 55.4 15 29.6 2.426 0.1661 0 0 0

Hampton Organic Soybean Field 1.02 48.6 17.8 33.6 1.681 0.0878 1 0 0

Hampton Organic Soybean Margin 1.25 55.1 14 30.9 1.833 0.09 0 0 0

***^a^*** CFUs: Log10 *Metarhizium* *anisopliae* s.l. colony forming unit (CFU) g^-1^ soil

*^b^* % C: Percent organic carbon in soil sample

*^c^* % N: Percent total nitrogen in soil sample

*^d^* Tillage: Binomial values for practice. 1 = practice applied; 0 = practice not applied. Same values apply for “Org. F” (organic fertilizer) and “Herbicides” columns

**2011 Field Survey (continued)**

**Site Practice Crop Treatment CFUs*^a^* % Sand % Clay % Silt % C*^b^* % N*^c^* Tillage*^d^* Org. F Herbicides**

Carroll Conventional Corn Field 0.00 2.5 28.5 69 4.066 0.228 1 0 1

Carroll Conventional Corn Margin 3.72 1.7 31.5 66.8 2.391 0.1647 0 0 0

Carroll Conventional Soybean Field 0.00 2 29.5 68.5 2.599 0.2085 1 0 1

Carroll Organic Corn Field 3.37 5.1 31.4 63.5 2.115 0.1643 1 1 0

Carroll Organic Corn Margin 3.42 1.8 29.5 68.7 3.54 0.2297 0 0 0

Carroll Organic Soybean Field 3.88 5.7 27 67.3 2.315 0.1593 1 1 0

Carroll Organic Soybean Margin 2.37 13.6 27.7 58.7 2.164 0.1502 0 0 0

Sioux Center Conventional Corn Field 0.00 2.2 29.8 68 2.86 0.1767 1 0 1

Sioux Center Conventional Corn Margin 0.00 3.6 30.7 65.7 3.457 0.2295 0 0 0

Sioux Center Conventional Soybean Field 1.03 2.2 29.3 68.5 2.482 0.1897 0 0 1

Sioux Center Conventional Soybean Margin 1.03 4.8 28.7 66.5 3.026 0.1909 0 0 0

Sioux Center Organic Corn Field 3.44 2.9 29.9 67.2 2.508 0.1419 1 0 0

Sioux Center Organic Corn Margin 4.04 31.6 20.5 47.9 3.651 0.2148 0 0 0

Sioux Center Organic Soybean Field 1.04 2.6 33.3 64.1 2.376 0.1615 1 0 0

Sioux Center Organic Soybean Margin 0.00 3.6 31.3 65.1 4.045 0.3009 0 0 0

Sutherland Conventional Corn Field 0.00 2.1 35.2 62.7 2.826 0.1823 1 0 1

Sutherland Conventional Corn Margin 3.74 2 37.4 60.5 3.087 0.1866 0 0 0

Sutherland Conventional Soybean Field 1.04 1.8 34.8 63.4 3.252 0.1504 1 0 1

Sutherland Conventional Soybean Margin 1.04 1.4 37.1 61.5 3.967 0.226 0 0 0

Sutherland Organic Corn Field 2.28 2.3 31.6 66.1 3.448 0.2118 1 1 0

Sutherland Organic Corn Margin 2.23 2.6 29.7 67.7 3.628 0.2273 0 0 0

Sutherland Organic Soybean Field 3.32 3.7 28.1 68.3 2.678 0.1556 0 0 0

Sutherland Organic Soybean Margin 2.07 3.7 32 64.3 3.15 0.2042 0 0 0

***^a^*** CFUs: Log10 *Metarhizium* *anisopliae* s.l. colony forming unit (CFU) g^-1^ soil

*^b^* % C: Percent organic carbon in soil sample

*^c^* % N: Percent total nitrogen in soil sample

*^d^* Tillage: Binomial values for practice. 1 = practice applied; 0 = practice not applied. Same values apply for “Org. F” (organic fertilizer) and “Herbicides” columns
