# Supplementary material for: Abundance of Soil-Borne Entomopathogenic Fungi in Organic and Conventional Fields in the Midwestern USA with an Emphasis on the Effect of Herbicides and Fungicides on Fungal Persistence
Source: PLoS One. 2015 Jul 20;10(7):e0133613. doi: 10.1371/journal.pone.0133613 (PMC4507996; doi:10.1371/journal.pone.0133613)
Supplement: S2 Table — (DOCX) [file pone.0133613.s002.docx]

**2012 Field Survey**

**Site Practice Crop Treatment CFUs*^a^* % Sand % Clay % Silt % C*^b^* % N*^c^* Tillage*^d^* Org. F Herbicides**

Iowa Falls Conventional Corn Field 0.00 35.7 23 41.2 1.899 0.1641 1 1 1

Iowa Falls Conventional Corn Margin 0.00 33.4 23.9 42.7 3.816 0.3376 0 0 0

Iowa Falls Conventional Soybean Field 0.99 47.5 20.8 31.7 1.838 0.1695 1 0 1

Iowa Falls Conventional Soybean Margin 3.32 49.9 19.3 30.8 2.542 0.2312 0 0 0

Iowa Falls Organic Corn Field 1.99 25.8 29.5 44.7 3.391 0.2806 1 1 0

Iowa Falls Organic Corn Margin 0.00 41.4 22.1 36.5 3.669 0.2893 0 0 0

Iowa Falls Organic Soybean Field 0.00 53.2 20.3 26.5 1.681 0.153 1 1 0

Iowa Falls Organic Soybean Margin 3.17 48.9 19.3 31.8 1.879 0.1828 0 0 0

Kalona Conventional Corn Field 2.21 3.2 18.6 78.2 2.108 0.2294 1 1 1

Kalona Conventional Corn Margin 1.95 3.8 21.2 75 1.191 0.1027 0 0 0

Kalona Conventional Soybean Field 0.00 3.3 18.4 78.3 1.66 0.1679 0 0 1

Kalona Conventional Soybean Margin 3.74 4.3 20.3 75.4 2.21 0.19 0 0 0

Hampton Conventional Corn Field 0.98 58.4 15.3 26.3 1.267 0.1459 1 0 1

Hampton Conventional Corn Margin 0.00 2.2 33.2 64.6 0.9547 0.0719 0 0 0

Hampton Conventional Soybean Field 4.14 49.4 20.1 30.4 1.353 0.1222 1 0 1

Hampton Conventional Soybean Margin 0.98 51.4 19.5 29.1 1.67 0.1478 0 0 0

Hampton Organic Corn Field 2.17 50.6 17.3 32.1 1.614 0.1509 1 1 0

Hampton Organic Corn Margin 0.95 52.1 17.4 30.5 1.697 0.1238 0 0 0

Hampton Organic Soybean Field 1.95 53.6 15.9 30.5 1.588 0.1257 1 1 0

Hampton Organic Soybean Margin 0.98 59.1 14.8 26 1.285 0.1062 0 0 0

***^a^*** CFUs: Log10 *Metarhizium* *anisopliae* s.l. colony forming unit (CFU) g^-1^ soil

*^b^* % C: Percent organic carbon in soil sample

*^c^* % N: Percent total nitrogen in soil sample

*^d^* Tillage: Binomial values for practice. 1 = practice applied; 0 = practice not applied. Same values apply for “Org. F” (organic fertilizer) and “Herbicides” columns

**2012 Field Survey (continued)**

**Site Practice Crop Treatment CFUs*^a^* % Sand % Clay % Silt % C*^b^* % N*^c^* Tillage*^d^* Org. F Herbicides**

Carroll Conventional Corn Field 0.00 2.1 29.4 68.5 2.382 0.2461 0 0 1

Carroll Conventional Corn Margin 0.00 1.5 30.7 67.7 4.139 0.368 0 0 0

Carroll Conventional Soybean Field 2.27 3.9 24.5 71.6 3.26 0.3003 0 0 1

Carroll Conventional Soybean Margin 0.00 2.6 26.2 71.2 2.067 0.2053 0 0 0

Carroll Organic Soybean Field 1.22 4.5 30.4 65.2 2.416 0.2417 0 1 0

Carroll Organic Soybean Margin 0.00 5.7 29.8 64.5 2.905 0.2296 0 0 0

Sutherland Conventional Corn Field 0.00 1.8 37 61.2 3.216 0.2779 1 0 1

Sutherland Conventional Corn Margin 0.00 1.9 30.6 67.5 4.391 0.3698 0 0 0

Sutherland Conventional Soybean Field 0.00 2.7 32.1 65.2 3.031 0.267 1 0 1

Sutherland Conventional Soybean Margin 1.98 2.6 33.2 64.2 4.507 0.3739 0 0 0

Sutherland Organic Corn Field 0.00 3.9 29.4 66.7 2.936 0.2749 1 1 0

Sutherland Organic Corn Margin 3.21 3.8 27.5 68.7 4.792 0.4315 0 0 0

Sutherland Organic Soybean Field 0.00 2.6 32.5 64.9 3.47 0.3139 1 0 0

Sutherland Organic Soybean Margin 0.99 3.2 28.1 68.7 3.936 0.3589 0 0 0

Sioux Center Conventional Corn Field 1.14 2.4 27.5 70.1 2.745 0.2541 1 0 1

Sioux Center Conventional Corn Margin 3.14 6.4 20.1 73.6 3.406 0.3102 0 0 0

Sioux Center Conventional Soybean Field 0.00 3 29.8 67.2 3.103 0.2827 1 0 1

Sioux Center Conventional Soybean Margin 0.00 4.4 29.9 65.7 3.666 0.3225 0 0 0

Sioux Center Organic Soybean Field 1.00 3.2 33.3 63.5 2.521 0.2448 1 0 0

Sioux Center Organic Soybean Margin 4.70 21.6 19.3 59.1 3.949 0.3413 0 0 0

***^a^*** CFUs: Log10 *Metarhizium* *anisopliae* s.l. colony forming unit (CFU) g^-1^ soil

*^b^* % C: Percent organic carbon in soil sample

*^c^* % N: Percent total nitrogen in soil sample

*^d^* Tillage: Binomial values for practice. 1 = practice applied; 0 = practice not applied. Same values apply for “Org. F” (organic fertilizer) and “Herbicides” columns
